# A Rapid Fluorescence Sensor for the Direct Quantification of Rongalite in Foodstuffs

**DOI:** 10.3390/foods11172650

**Published:** 2022-09-01

**Authors:** Hongfang Li, Jie Chen, Baowei Huang, Lingwei Kong, Feifei Sun, Lin Li, Chuanyi Peng, Huimei Cai, Ruyan Hou

**Affiliations:** 1 State Key Laboratory of Tea Plant Biology and Utilization, College of Tea and Food Science & Technology, Anhui Agricultural University, Hefei 230036, China; 2 Animal-Derived Food Safety Innovation Team, College of Animal Science and Technology, Anhui Agricultural University, Hefei 230036, China

**Keywords:** rongalite, fluorescence sensor, rapid detection, foodstuffs

## Abstract

Rongalite was reported illegally used as a food additive for bleaching purposes and improving the tenderness of foodstuffs, which may endanger public health. At present, rongalite was mostly detected by indirect methods via derivatization or determining its decomposition products. In this study, we developed a new fluorescence sensor for the direct quantification of rongalite based on the principles: (1) dopamine reacts with resorcinol and generates strong fluorophore (azamonardine); (2) rongalite could inhibit the production of fluorophores and then result in lower fluorescence intensity. Hence, the rongalite concentration was inversely proportional to fluorescence intensity of fluorophore. Several crucial reaction conditions of fluorescence sensor were further optimized, such as dopamine and resorcinol concentration, pH values, and reaction time. Under the optimal conditions, the limit of detection of fluorescence sensor was 0.28–0.38 μg/g in vermicelli, wheat and rice powder samples, exhibiting almost 3.5-fold improvement compared to that of lateral flow immunoassay. Moreover, the detection time was substantially decreased to 20 min. The recoveries in spiked samples were 80.7–102.1% with a coefficient of variation of less than 12.6%. In summary, we developed a direct, high throughput, selective and accurate fluorescence sensor that poses a promising application for the rapid detection of rongalite in foodstuffs.

## 1. Introduction

Rongalite, also known as sodium formaldehyde sulfoxylate, is a versatile and commercially available reagent, which is typically used as an industrial bleaching agent for vat dyeing as well as a green reagent in organic synthesis [1,2,3]. Although rongalite possesses diverse applications, its addition to food as a decolourant is prohibited. This is because the unstable rongalite in acidic solution and the heated condition is decomposed in equimolar amounts into formaldehyde and sodium bisulfite [4,5], both of which are serious threats to human health. As one of the decomposition products, formaldehyde plays a key role in food preservation but is associated with pain, nausea, dermatitis, central nervous system injury, and carcinogenic effects when the human body ingests formaldehyde in excessive quantities [6,7]. Sodium bisulfite, another decomposition product, is used as a color stabilizer and antioxidant in the food industry, however, it may generate harmful gas such as H_2_S and SO_2_ under certain conditions [8]. Rongalite possesses similar physical and chemical properties to both formaldehyde and sodium bisulfite, resulting in the motive for the abusive and substitutional use of rongalite in the food processing industry. As reported, the rongalite was illegally used as a food additive for bleaching purposes and to improve the tenderness of wheat powder, rice powder, tofu, vermicelli, and other foodstuffs, which caused adverse effects on human health in different countries [9,10]. Thus, it is urgent and necessary to develop a reliable, rapid, and sensitive detection method for rongalite analysis in food samples.

Until now, the analysis method for rongalite includes indirect and direct methods. The indirect methods generally defined the decomposition products (formaldehyde and sodium bisulfite) as detection makers rather than rongalite directly, maybe leading to ambiguous and inaccurate detection results [11,12]. In addition, the indirect methods mainly consisted of instrumental methods such as capillary electrophoresis [13] and high-performance liquid chromatography [14], which suffer from sophisticated instrumentation and tedious sample preparation. Hence, the indirect methods were not a promising method for accurate and convenient analysis of rongalite. Recently, several direct methods that adopted rongalite itself as the detection maker were reported as alternative methods for the rapid detection of rongalite. For instance, based on a rongalite-specific aptamer, Li et al. established a sandwich lateral flow strip (LFIA) assay with the limit of detection (LOD) of 1.0 μg/mL [15]; Jing et al. developed a sandwich-type enzyme-linked immunosorbent assay (ELISA) with the LOD of 0.57 ng/mL (Table S1) [16]. Although the aptamer-participated methods were rapid and sensitive, the key reagent aptamer with high specificity and affinity was hard to be prepared. Recently, Abubakar et al. reported a fast but low-flux electrochemiluminescence (ECL) method for the detection of rongalite in foodstuffs with a LOD of 0.07 μg/mL (Table S1) [9]. Nowadays, with the increasing demand for quicker and higher throughput screening of rongalite, ongoing effort should be focused on developing new and generic detection methods, and the rongalite residue in food should be timely assessed.

Fluorescence-based sensing platforms were a promising alternative system for achieving rapid implementation, sensitive and high-throughput detection of chemical contaminants [17,18]. In fluorescent analysis, fluorophores serve as an important signal reporter and achieve the goal of quantitative analysis through “turn on” or “turn off” responses. Recently, the fluorophores that were prepared through a one-step bimolecular reaction attracted lots of attention and were used in the analysis field [19,20]. For example, Acuna et al. described a strong yield fluorophore (azamonardine product) that was prepared by a simple reaction of resorcinol and catecholamines, and then the fluorophore could be used for analytical and imaging techniques [21]. Based on the above study, some researchers employed dopamine (or resorcinol) as the catalytic product of tyrosinase (or alkaline phosphatase), and then added the incorporating substrate to induce a fluorescence response [22,23,24,25].

Inspired by the above research, we propose that the introduction of a strong reductant (rongalite) could inhibit the introduction of fluorophores, achieving the quantification of rongalite in foods. Herein, we explored the fluorescence intensity response between fluorophore and diversity concentrations of rongalite and construct a simple on-site detection system for the rapid detection of rongalite in vermicelli, wheat and rice powder. This research provides a new strategy for the determination of rongalite in foods quality control.

## 2. Materials and Methods

### 2.1. Materials and Apparatus

Rongalite, dopamine hydrochloride and resorcinol were purchased from Macklin Company (Shanghai, China). Formaldehyde solution (37–40%) was obtained from J &K Scientific (Beijing, China). Glucose, sucrose, glutamate, prolamin and Ca^2+^ were supplied by Aladdin Industrial Corporation (Shanghai, China). Sodium carbonate, sodium hydroxide and hydrochloric acid were purchased from Sinopharm Chemical Reagent Co., Ltd. (Beijing, China). All the reagents were analytical grade.

Sterile syringe filters (0.45 μm) were obtained from Merck (Merck Millipore, Burlington, MA, USA). White opaque microplates were supplied by Corning Ltd. (Corning Life Science, Hartford, NY, USA). Ultra-pure water was used in all aqueous solution and was produced by Millipore purification system (MilliQ gradient A10, Millipore, Bedford, MA, USA). Fluorescence excitation and emission spectra, absorption spectra, optical density, and fluorescence intensity were measured using a microplate reader (SpectraMax M2e, Molecular Devices, San Jose, CA, USA). The pH values were measured by a pH meter supplied by Thermo Fisher Scientific Inc. (Orion 4 Star, Beverly, MA, USA). The mass spectrum analysis was performed on the electrospray ionization (ESI)-TRIPLE QUAD 5500 (AB SCIEX, Framingham, MA, USA.) in the negative ion mode.

### 2.2. Characterization of the Fluorescence Sensing Platform

Dopamine and resorcinol were dissolved in ultra-pure water at the concentrations of 50 and 20 μM, respectively. The dopamine (30 μL) and resorcinol (30 μL) solution were injected into the microplates, followed by the addition of 200 μL NaOH solution (pH 9.5) or rongalite standard solution (5 μg/mL, in NaOH solution, pH 9.5). The mixture solution was incubated in a metal bath at 45 °C for 20 min before detection. The absorption spectrum, fluorescence spectrum, optical density (OD), and fluorescence intensity of four solutions (dopamine, resorcinol, mixture solution of dopamine and resorcinol, mixture solution of dopamine, resorcinol and rongalite, 260 μL) were measured using a microplate reader. Note that dopamine hydrochloride was abbreviated to dopamine in this paper. 

### 2.3. Dopamine and Resorcinol Concentration-Response

The checkerboard assay and two-point (0 and 0.25 μg/mL rongalite) competitive format was adopted to study the optimal concentrations of dopamine and resorcinol solution. Briefly, the concentrations of dopamine solution were 5, 10 and 50 μM with the resorcinol solution ranging from 1.25 to 40 μM. Then, the fluorescence intensity (λ_ex_, 420 nm, λ_em_, 465 nm) was measured, and the inhibition ratio (Equation (1)) at different concentrations was recorded to evaluate the response tendency.

Inhibition ratio (%) = (F_0_ − F_1_)/F_0_ × 100% (1)

where F_0_ and F_1_ are the fluorescence intensity of the wells in the absence of rongalite and in the presence of rongalite, respectively.

### 2.4. Reaction Conditions Optimization

#### 2.4.1. pH Values Optimization

The NaOH solution at different pH values (from 6 to 12, with a concentration gradient of 1) was prepared to explore the relationship between fluorescence intensity and pH values. The microplates were incubated at 45 °C for 20 min, and then the fluorescence spectrum (λ_ex_, 420 nm) and fluorescence intensity (λ_ex_, 420 nm, λ_em_, 465 nm) were measured, separately.

#### 2.4.2. Carbonate Concentration Optimization

The carbonate solution was more stable than the NaOH solution, thus we further optimized the carbonate concentration at 0, 5, 7.5, 10, 12.5, 15, 17.5 and 20 mM. The detailed experiment procedure was similar to the “Dopamine and resorcinol concentration-response” section, except for replacing the NaOH solution with carbonate solution. 

#### 2.4.3. Reaction Time Optimization

The reaction time of the fluorescence sensing system was optimized to evaluate whether the reactions reached equilibrium. In total, six time points from the reaction process were selected (5, 10, 15, 20, 30 and 40 min), and the corresponding fluorescence spectrum and intensity were measured.

### 2.5. Fluorescence Sensing Platform Development

Under the optimal conditions, various rongalite solutions (sample extracts or other related compounds, 200 μL) at consecutive concentrations were added to the mixture reaction system of dopamine (30 μL) and resorcinol (30 μL) and incubated for 20 min. The fluorescence intensity (λ_ex_, 420 nm, λ_em_, 465 nm) was measured and the sigmoidal curve was fitted using a four-parameter logistic equation (Equation (2)) with the assistance of OriginPro 8.0 (Northampton, MA, USA). Some agents were evaluated to explore the selectivity of our fluorescence sensing system at 0, 0.25 and 10 μg/L, such as formaldehyde, sodium bisulfite, glucose, sucrose, glutamate, prolamin and Ca^2+^.

Y = (A − B)/[1 + (X/C)^D^] + B (2)

where A and B represent the responses at high and low asymptotes of the curve, C stands for the concentration of the analytes resulting in 50% inhibition, D represents the slop factor, and X is the calibration concentration.

### 2.6. Sample Preparation and Method Validation

#### 2.6.1. Sample Preparation

The sample preparations of vermicelli, wheat and rice powder were operated according to the previous study with some modifications [16]. 1.0 g wheat or rice powder (vermicelli was ground into powder) was dispersed in 5 mL carbonate solution (2 mM, pH 10), and the mixture was maintained for 10 min under ultra-sonication (40 °C). After 15 min of centrifugation at 10,000 rpm, the supernatant solution (1.0 mL) was collected and filtered with sterile syringe filters (0.45 μm), diluted with carbonate solution 4 times, and then subsequently submitted to the fluorescence sensing platform for the rongalite detection.

#### 2.6.2. Limit of Detection

Negative samples were supplied and verified by Anhui Public Inspection Research Institute Co., Ltd. (Hefei, China). The LOD was defined as the concentration corresponding to the average fluorescence intensity of 20 negative samples plus 3 times the standard deviation (three independent experiments) [26,27].

#### 2.6.3. Method Validation

Three samples, including vermicelli, wheat and rice powder were spiked with rongalite at different concentrations. After sample pretreatment, the samples were detected using the fluorescence sensing platform. Subsequently, the recovery ratios and variations in intra- and inter-assays were calculated.

## 3. Results and Discussion

### 3.1. Assay Principle for the Fluorescence Sensing Platform

**Scheme 1:**
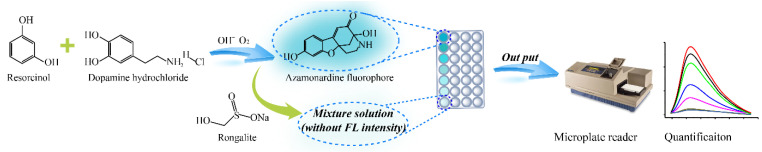
Reaction mechanisms of fluorescence sensor for rongalite detection. Resorcinol reacts with dopamine and produces blue fluorophore (azamonardine) under alkaline conditions. The fluorescence intensity of azamonardine sharply decreased with the addition of rongalite, and the standard curve is constructed based on fluorescence intensity. Subsequently, the rongalite concentration in foodstuffs is measured via microplate reader.

**Figure 1:**
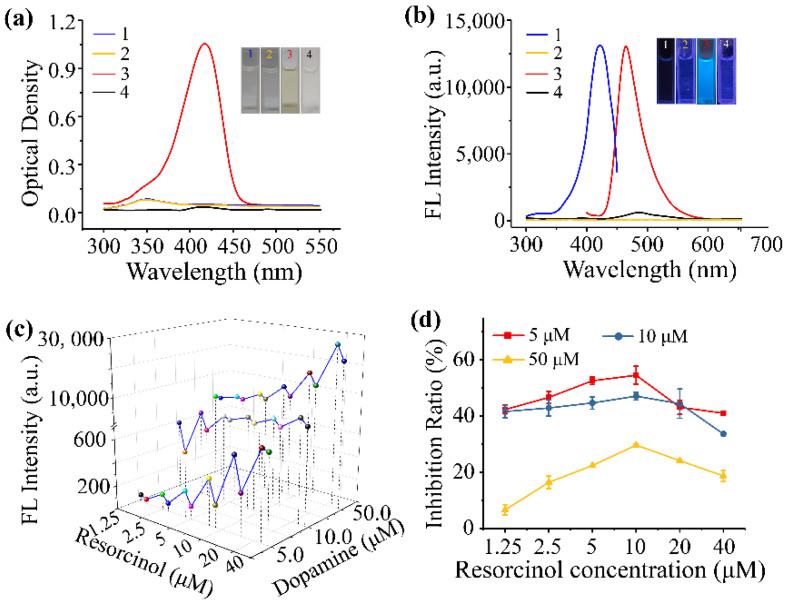
The (**a**) UV-vis absorption spectra and (**b**) fluorescence spectra of four solutions (named by 1, 2, 3 and 4), (**c**) fluorescence intensity and (**d**) inhibition ratio for dopamine and resorcinol concentration-response. Four numbers (1–4) inserted in the figures means dopamine, resorcinol, the mixture solution of dopamine and resorcinol, the mixture solution of dopamine, resorcinol and rongalite, respectively.

According to the previous reports, under the alkaline condition, the dopamine could react with resorcinol within several minutes, producing in situ azamonardine fluorophores via a nucleophilic attack (shown in Scheme 1) [25]. In this research, we introduced a rongalite solution in the reaction system, and a surprising decreased optical density and fluorescence intensity were observed in Scheme 1. In detail, the absorption peaks of dopamine and resorcinol were both 351 nm (Figure 1a). A new absorption peak for the reaction product of dopamine and resorcinol was 415 nm with an optical density of 1.06, and its optical density was weakened with the addition of rongalite. Subsequently, the fluorescence signal of the above solutions was further characterized. The maximum excitation and emission wavelengths of the product solution were centered around 420 nm and 465 nm (Figure 1b). It is obvious that the fluorescence intensity (1,3125) decreased to 625 since the addition of rongalite. Thus, the in situ system could be used for the quantification of rongalite in samples. We finally selected the fluorescence system for the subsequent research rather than optical signals because the fluorescence system has higher sensitivity owing to its high signal-to-noise (S/N) ratio. 

We hypothesize that there existed two possibilities for the addition of rongalite to reduce the fluorescence intensity: (1) the rongalite acts as a protective agent and prevents the oxidizing of dopamine or reduce the reaction product, resulting in the decreased generation of azamonardine fluorophores; (2) the rongalite undergoes a reduction reaction with dopamine or resorcinol, which reduce the concentration of reaction substrate and inhibited the production of fluorophores. To illustrate the principle of the in situ fluorescence assays, the chemicals in the reaction system were characterized using HPLC-MS. The dopamine hydrochloride and resorcinol exhibited ion peaks at 188.0 and 109.0 (m/z, [M-H]^−^) (Figure S1a,b). The ion peaks of rongalite couldn’t be found at 117 (m/z, [M-H]^−^), because of the high-temperature vaporization of rongalite. As shown in Figure S1c, the intense ion peak at 258.1 (m/z, [M-H]^−^) was attributed to azamondardine (reaction product of dopamine and resorcinol, without purification), which was consistent with the previous research [25]. The intensity of azamondardine sharply decreased from 8.9 × 10^7^ to 4.0 × 10^3^, when rongalite was presented in the reaction system (Figure S1d). Moreover, we observed that the mixture of dopamine and rongalite, or resorcinol and rongalite wouldn’t produce a new chemical reaction. This was because the ion peaks intensity of dopamine and resorcinol remained almost unchanged, and moreover there didn’t exist new ion peaks (Figure S2a,b). Herein, our first supposition, whereby rongalite served as a protective agent and prevented the yielding of azamonardine fluorophores, was true and proved to be a new strategy for the detection of rongalite.

### 3.2. Dopamine and Resorcinol Concentration–Response

Dopamine and resorcinol concentration–response is closely related to the sensitivity of the fluorescence sensing platform. In theory, the higher concentration of dopamine and resorcinol would generate bright fluorescence, resulting in faint competitive affection of rongalite and lower sensitivity. On contrary, rongalite at low concentrations could inhibit the yield of fluorescence when a lower concentration of reactant was used. In this case, the fluorescence sensor possesses excellent sensitivity but is unstable due to the obvious variation coefficient of lower fluorescent intensity. Here, we explored the dopamine and resorcinol concentration–response by analyzing the variation of fluorescent intensity via a two-point competitive format. 

The fluorescent intensity heightened evidently with the concentration of dopamine varying from 5 to 50 μM and resorcinol changing from 1.25 to 40 μM, due to the reaction product (azamonardine fluorophores) increased. However, the corresponding fluorescent intensity also increased in the presence of rongalite (Figure 1c). It is hard to select the superior point of dopamine and resorcinol concentration–response. Subsequently, the inhibition ratio was evaluated and described in Figure 1d. We found that dopamine concentration has an obvious effect on the inhibition ratio at the same resorcinol concentration. The lower inhibition ratio (6.7–29.7%) was observed when dopamine concentration was 50 μM. There was no significant difference in terms of inhibition ratio (44.9–52.2%) when resorcinol concentration ranged from 2.5 to 20 μM and dopamine concentrations were 5 and 10 μM. Finally, the 5.0 μM and 10.0 μM were separately selected as the optimal response concentrations for dopamine and resorcinol, considering the appropriate fluorescence intensity (388) and the highest inhibition ratio values (52.2%). 

### 3.3. Optimization of Reaction pH, Carbonate Concentration and Response Time

Three experiment parameters, including reaction pH, carbonate concentration and response time, were further optimized for improving the stability, sensitivity and saving the reaction time of the fluorescence sensor. 

**Figure 2:**
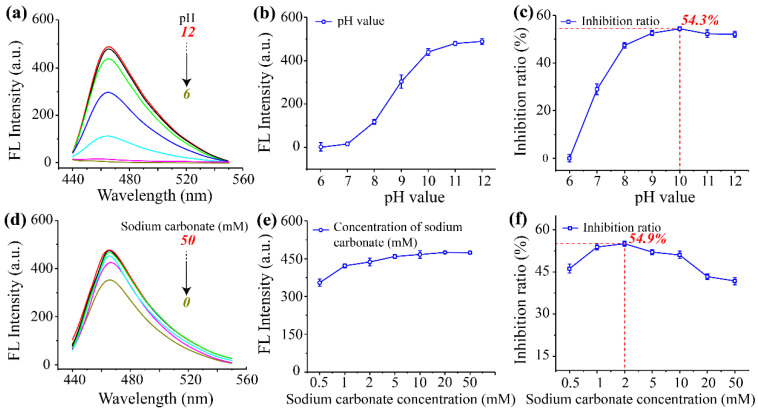
The optimization of pH values and sodium carbonate concentrations. (**a**) fluorescence spectra, (**b**) fluorescence intensity and (**c**) inhibition ratio at different pH values, (**d**) fluorescence spectra, (**e**) fluorescence intensity and (**f**) inhibition ratio at different sodium carbonate concentrations.

The pH values were a vital parameter for the reaction system because the formation of azamonardine relies on a rigid alkaline condition. Here, we investigated the effect of pH values on fluorescence intensity under the optimal dopamine and resorcinol concentration-response. The fluorescence intensity raised following the increase of pH values (Figure 2a), and its corresponding values at 465 nm were exhibited in Figure 2b. Briefly, the fluorescence intensity was close to 0, when the pH values were 6 and 7. This result indicated the chemical reaction between dopamine and resorcinol was hardly to occur under acidity or neutral conditions, which was consistent with previous research [20]. Meanwhile, the inhibition ratio was low (−2.2% to 28.9%, Figure 2c). In the absence of rongalite, the fluorescence intensity increased from 117 to 488 when the pH values ranged from 8 to 12; however, the fluorescence intensity in the presence of rongalite (0.25 μg/mL) also increased at the same time (Figure S3). Thus, the inhibition ratios increased followed by a decrease. A higher inhibition ratio usually means higher sensitivity. Hence, 10 was selected as the optical reaction pH value for the fluorescence sensor with an inhibition ratio of 54.3%.

Generally, the buffer solution plays a vital role in fluorescence analysis, which furnishes stable and accurate detection conditions by sustaining the pH and ionic strength at constant values [28]. In this research, the carbonate buffer solution served as the system buffer solution, considering it was the frequently used alkaline buffer solution. We hypothesize the ionic strength at different concentrations might interfere with the stability of the fluorescence signal. Here, we explored the affection of ionic strength concentration of carbonate buffer (pH 10) on fluorescence intensity and inhibition ratio. The discrete degree of fluorescence signal was low at different ionic strength concentrations, illustrating the ionic strength did not affect the stability of the fluorescent signal (Figure 2d,e and Figure S4). However, a slight variation in fluorescence intensity and inhibition ratio was observed, which confused us. Finally, the ionic strength at 2 mM contributed to the higher inhibition ratio (54.9%) and was selected as the excellent concentration (Figure 2f). 

**Figure 3:**
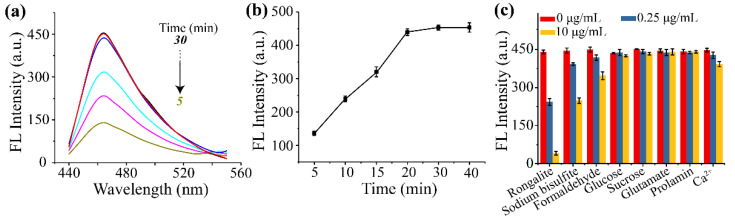
The (**a**) fluorescence spectra, (**b**) fluorescence intensity of the fluorescence sensor with the time ranged from 5 to 40 min. (**c**) The selectivity of the fluorescence sensor.

The fluorescence signal of the product will continue to rise or be unstable if the reaction has not reached equilibrium. Consequently, we evaluated the response time to develop an accurate and constant fluorescence sensor. As shown in Figure 3a,b, the fluorescence signal sharply increased from 136 to 438 between 5 min and 20 min and tended to be balanced at 20 min. Aiming to improve detection efficiency, we choose 20 min as the optical response time. Generally, a rapid detection method could provide a report in <25 min [29,30]. Therefore, our fluorescence sensor has obvious advantages in terms of saving time.

### 3.4. Development of Fluorescence Sensor

**Figure 4:**
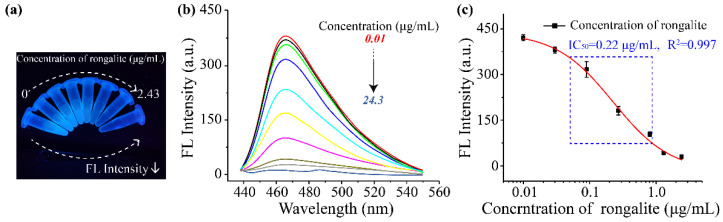
(**a**) Fluorescence photos and (**b**) fluorescence spectra of the fluorescence sensor exposed on a series of rongalite concentrations. (**c**) The standard curve of the quantification of rongalite.

Under the optimal conditions, a series of rongalite concentrations were prepared for construct a standard curve, i.e., 0, 0.01, 0.03, 0.09, 0.27, 0.81, 1.30 and 2.43 μg/mL. The fluorescence intensity faded with the increase of the rongalite concentration in the buffer solution (Figure 4a,b). Subsequently, a standard curve was fitted using a four-parameter logistic equation (Figure 4c) with the half inhibitory concentration of 0.22 μg/mL (R^2^, 0.993), and its linear range was 0.05 μg/mL to 0.90 μg/mL (marked with the blue outline).

We explored the selectivity of fluorescence sensor by analyzing some reducing agents (formaldehyde, sodium bisulfite), and matrix factors (glucose, sucrose, glutamate, prolamin, Ca^2+^). As shown in Figure 3c, although formaldehyde and sodium bisulfite displayed a response to the fluorescence intensity, the response concentration that made the signal decrease in half was higher than 10 μg/mL. Hence, these interfering factors could be recognized as the negligible response. Other interfering factors would not generate the signal response, which indicated the fluorescence sensor was selective enough for the rongalite detection.

Compared with the instrumental analysis, a simple and convenient sample preparation method was a crucial section for rapid detection [31,32]. Generally, for combined forms of analytes and samples, the analytes in samples were extracted using organic solvent followed by dilution; otherwise, they were extracted with buffer solution for dissociated forms of analytes and samples or analytes with higher water solubility. In this research, the carbonate buffer solution (pH 10, 2 mM) was selected to extract the rongalite in samples. The matrix effect of the extract was expected to be removed by dilution and was explored in two steps. Firstly, the extraction solution of samples was diluted with carbonate solution for 2, 4 and 8-fold, and then its fluorescence intensity was measured using the fluorescence sensing platform. Owning to the matrix factors could interfere with the trigger of fluorescence signal, hence, the lowest dilution corresponding to the fluorescence intensity similar to that in buffer solution was preliminarily selected as the point eliminating the matrix effect of samples. Compared with the fluorescence intensity in buffer solution (in the absence of rongalite), 2-fold dilution of extraction solution exhibited obvious matrix effect along with the decreased signal (Figure S5). Nevertheless, 4- and 8-fold dilution could avoid the matrix effect. Obviously, a 4-fold dilution appeared to avoid the matrix effect. Secondly, the matrix calibration curve was constructed via spiking rongalite at different concentrations in the 4-fold extraction solution. As shown in Figure S6, all the extraction calibration curves were in good agreement with the standard curve in buffer solution, which indicated the 4-fold dilution eliminated the matrix effect. Eventually, a 4-fold dilution of extraction solution was selected as the preferred preparation method for samples considering the higher sensitivity of the fluorescence sensor.

Detailed parameters about average fluorescence intensity of 20 negative samples and standard deviation were displayed in Table S2. Subsequently, the calculation results were submitted to the standard curve and obtained the LOD. Thus, the LOD was measured after sample preparation, and was calculated as 0.38, 0.28 and 0.32 μg/g for vermicelli, wheat and rice powder, and the linear range was about 1–18 μg/g.

Despite possessing sensitive properties, the reported indirect assays generally quantified rongalite via derivatization or determining its decomposition products [11]. In contrast, our research could achieve the goal of direct and rapid detection of rongalite. Here, we summarized several direct and rapid detection methods for rongalite in foodstuffs and compared them with our fluorescent assay (Table S1). The proposed fluorescence sensor displayed an excellent performance in LOD (0.28–0.38 μg/g) compared with that of the lateral flow immunoassay (1 μg/mL) but exhibited higher LOD compared with other assays. Actually, LOD is not the only criterion for evaluating assay performance; therefore, other parameters should be taken into accounts, such as the required materials and linear range for the assay and the pretreatment procedure for samples. Among the assays, the ECL method has the widest linear range (4.7–118 μg/mL) [9]. It was also observed that the dilution factor was 40, which means the ECL method was susceptible to the matrix effect of samples and the stability of the detection result might be interfered. Comparatively, our proposed fluorescent sensor possessed higher matrix tolerance with a dilution factor of 20. The reported ELISA displayed an excellent LOD of 0.57 ng/mL. However, the experimental procedure was complex and the operation time was 110 min. Compared with ELISA, our fluorescent assay possessed the advantage in terms of less time (20 min), and saved 5.5-fold time. Actually, ELISA and LFIA both required aptamer as the recognition material. As reported, aptamer was usually acquired through multi-round exponential enrichment, its screening procedure being full of considerable workload. Preparing two aptamers to recognize different sites of the small molecule was especially a challenge. Thus, the ELISA and LFIA for quantification of rongalite based on aptamer both need a particular material, which might restrict its wide application in foodstuffs safety testing. In contrast, the fluorescent assay in this study adopted the traditional chemicals (dopamine, resorcinol) as the substrate, and exhibited a satisfactory detection time, high throughput, sensitivity as well as accuracy, possessing irreplaceable properties.

### 3.5. Accuracy of the Fluorescence Sensor

As previously reported, there are two main approaches to verify the accuracy of the rapid detection method: (1) detecting real samples using both instrumental analysis methods and rapid detection methods simultaneously, and comparing the consistency or correlation of detection results; (2) spiking the analyte in negative samples at different concentrations for preparing positive samples and measuring the recovery ratio using rapid detection method [33,34]. In this research, the accuracy of the proposed fluorescence sensor was analyzed by recovery ratios and the variations in intra- and inter-assays. The rongalite-contaminated samples were prepared by spiking 1, 2, and 4 μg/g in vermicelli, wheat and rice powder samples, respectively. The recovery ratio was 80.7–102.1% with a coefficient of variation of less than 8.6% (Table 1), which illustrates the fluorescence sensor was a promising method for rapid detection of rongalite in samples.

## 4. Conclusions

In conclusion, we developed a fluorescence sensor for the detection of rongalite within 20 min. This assay was constructed by introducing the chemical reaction system of dopamine, resorcinol and rongalite, the higher concentration of rongalite could inhibit the production of azocine fluorophores and then result in lower fluorescence intensity. Hence, the rongalite concentration was inversely proportional to fluorescence intensity of fluorophore, this detection mechanism was firstly proposed by our team. The fluorescence sensor possessed the advantages of high throughput, easy operation and rapid, exhibited a LOD of 0.28–0.38 μg/g in vermicelli, wheat and rice powder samples, respectively. The recovery ratio was higher than 80.7% with a coefficient of variation of less than 12.6%, which could satisfy the accuracy of the rapid detection. The novelty of this research was that we constructed a new fluorescence sensor, which contributed to the superiority of higher sensitivity, easy operation, time-saving and low cost for the detection of rongalite. This fluorescence sensor could be a potential tool for the rapid measurement of rongalite in foodstuffs.

## Figures and Tables

**Table 1:** Recovery, coefficient of variation of vermicelli, wheat and rice powder samples using fluorescence sensor (*n* = 3).

Samples	Spiked	Intra-Assay		Inter-Assay	
	(μg/g)	Recovery	CV ^1^	Recovery	CV ^1^
		(%)	(%)	(%)	(%)
Vermicelli	1	80.7	12.6	82.3	12.1
	2	88.4	8.1	85.3	10.1
	4	92.5	7.3	90.6	6.9
Wheat powder	1	86.9	7.5	84.1	9.7
	2	95.3	10.4	88.7	8.3
	4	102.1	6.9	95.8	5.4
Rice powder	1	89.6	9.1	81.2	10.8
	2	95.4	4.2	87.7	8.6
	4	100.5	5.4	90.5	7.2

^1^ means the abbreviation of coefficient.

## Data Availability

Data is contained within the article or Supplementary Material.

## Supplementary Materials

The following supporting information can be downloaded at: https://www.mdpi.com/article/10.3390/foods11172650/s1, Figure S1: HPLC-MS of dopamine hydrochloride, resorcinol and mixture solution; Figure S2: HPLC-MS of the mixture of dopamine hydrochloride and rongalite, the mixture of resorcinol and rongalite; Figure S3: fluorescence intensity at different pH values; Figure S4: fluorescence intensity at different carbonate solution concentrations; Figure S5: matrix effect of vermicelli, wheat and rice powder for the fluorescence sensor; Figure S6: matrix calibration curve of vermicelli, wheat and rice powder; Table S1: an overview of the reported method for the rapid detection of rongalite; Table S2: limit of detection of three samples using fluorescence sensor.

## References

[1] Ali R. (2020). New dimensions in rongalite chemistry: The land of opportunities in organic synthesis and material sciences. ChemistrySelect.

[2] Aravindu K., Cloutet E., Brochon C., Hadziioannou G., Vignolle J., Robert F., Taton D., Landais Y. (2018). Poly(arylene vinylene) synthesis via a precursor step-growth polymerization route involving the ramberg-bäcklund reaction as a key post-chemical modification step. Macromolecules.

[3] Yemata T.A., Zheng Y., Ko Kyaw A.K., Wang X., Song J., Chin W.S., Xu J. (2020). Sodium formaldehyde sulfoxylate, an ionic-type, water-soluble reducing reagent to effectively improve seebeck coefficient of PEDOT:PSS film. Org. Electron..

[4] Kotha S., Khedkar P. (2012). Rongalite: A useful green reagent in organic synthesis. Chem. Rev..

[5] Makarov S.V., Horvath A.K., Silaghi-dumitrescu R., Gao Q. (2019). Reactivity of small oxoacids of sulfur. Molecules.

[6] Kamps J.J.A.G., Hopkinson R.J., Schofield C.J., Claridge T.D.W. (2019). How formaldehyde reacts with amino acids. Commun. Chem..

[7] Nowshad F., Islam M.N., Khan M.S. (2018). Concentration and formation behavior of naturally occurring formaldehyde in foods. Agric. Food Secur..

[8] Makarov S.V., Horvath A.K., Silaghi-dumitrescu R., Gao Q. (2016). Sodium Dithionite, Rongalite and Thiourea Oxides: Chemistry and Application.

[9] Abdussalam A., Yuan F., Ma X., Du F., Zholudov Y.T., Zafar M.N., Xu G. (2020). Tris(2,2′-bipyridine)ruthenium(II) electrochemiluminescence using rongalite as coreactant and its application in detection of foodstuff adulteration. J. Electroanal. Chem..

[10] Wang H., Cao X., Peng X., Hu C., Huang J., He H. (2020). Uncertainty evaluation of high performance liquid chromatographic determination of sodium formaldehyde sulfoxylate (rongalite) in wheat powder and rice powder. Food Sci..

[11] de Carvalho L.M., Schwedt G. (2005). Sulfur speciation by capillary zone electrophoresis. J. Chromatogr. A.

[12] Wu Z., Guo F., Huang L., Wang L. (2018). Electrochemical nonenzymatic sensor based on cetyltrimethylammonium bromide and chitosan functionalized carbon nanotube modified glassy carbon electrode for the determination of hydroxymethanesulfinate in the presence of sulfite in foods. Food Chem..

[13] Guo Q., Mu T., Wu Y., Yu X., Song J., Zhou J., Yang H. (2015). Determination of sodium formaldehyde sulfoxylate residues in wheat flour and bean starch noodles by capillary electrophoresis-contactless conductivity detection. Food Sci..

[14] Zhang Y., Pan X., Zhang L. (2017). Determination of sodium formaldehyde sulfoxylate in food by high-performance liquid chromatography. J. Food Saf. Qual..

[15] Li J., Jing L., Song Y., Zhang J., Chen Q., Wang B., Xia X., Han Q. (2018). Rapid detection of rongalite via a sandwich lateral flow strip assay using a pair of aptamers. Nanoscale Res. Lett..

[16] Jing L., Li J., Qin M., Song Y., Zhang J., Chen Q., Xia X., Han Q. (2018). Development and characterization of sandwich-type enzyme-linked aptamer assay for the detection of rongalite in food. Anal. Biochem..

[17] Goshisht M.K., Tripathi N. (2021). Fluorescence-based sensors as an emerging tool for anion detection: Mechanism, sensory materials and applications. J. Mater. Chem. C.

[18] Skorjanc T., Shetty D., Valant M. (2021). Covalent organic polymers and frameworks for fluorescence-based sensors. ACS Sens..

[19] Wang H., Zhou S., Guo L., Wang Y., Feng L. (2020). Intelligent hybrid hydrogels for rapidin situ detection and photothermal therapy of bacterial infection. ACS Appl. Mater. Inter..

[20] Zhang H., Xiao Y., Zhang X., Wang S. (2019). Modulating an in situ fluorogenic reaction for the label-free ratiometric detection of biothiols. Analyst.

[21] Acuña A.U., Álvarez-Pérez M., Liras M., Coto P.B., Amat-Guerri F. (2013). Synthesis and photophysics of novel biocompatible fluorescent oxocines and azocines in aqueous solution. Phys. Chem. Chem. Phys..

[22] El Sayed S., Pascual L., Licchelli M., Martínez-Máñez R., Gil S., Costero A.M., Sancenón F. (2016). Chromogenic detection of aqueous formaldehyde using functionalized silica nanoparticles. ACS Appl. Mater. Inter..

[23] Zhao J., Wang S., Lu S., Liu G., Sun J., Yang X. (2019). Fluorometric and colorimetric dual-readout immunoassay based on an alkaline phosphatase-triggered reaction. Anal. Chem..

[24] Zhao J., Wang S., Lu S., Bao X., Sun J., Yang X. (2018). An enzyme cascade-triggered fluorogenic and chromogenic reaction applied in enzyme activity assay and immunoassay. Anal. Chem..

[25] Zhao J., Liu G., Sun J., Wang Q., Li Z., Yang X. (2020). Dual-readout tyrosinase activity assay facilitated by a chromo-fluorogenic reaction between catechols and naphthoresorcin. Anal. Chem..

[26] Lu M., He Q., Zhong Y., Pan J., Lao Z., Lin M., Wang T., Cui X., Ding J., Zhao S. (2021). An ultrasensitive colorimetric assay based on a multi-amplification strategy employing Pt/IrO_2_@SA@HRP nanoflowers for the detection of progesterone in saliva samples. Anal. Methods-Uk.

[27] Qie Z., Liu Q., Yan W., Gao Z., Meng W., Xiao R., Wang S. (2019). Universal and ultrasensitive immunochromatographic assay by using an antigen as a bifunctional element and antialbumin antibody on a test line. Anal. Chem..

[28] Wang L., Wen L., Zhao L., Chao J., Tao F., Wang F., Li C. (2022). Development of fluorescence sensor and test paper based on molecularly imprinted carbon quantum dots for spiked detection of domoic acid in shellfish and lake water. Anal. Chim. Acta.

[29] Jayan H., Pu H., Sun D. (2020). Recent development in rapid detection techniques for microorganism activities in food matrices using bio-recognition: A review. Trends Food Sci. Tech..

[30] Wu H., Qian C., Wang R., Wu C., Wang Z., Wang L., Zhang M., Ye Z., Zhang F., He J. (2020). Identification of pork in raw meat or cooked meatballs within 20 min using rapid PCR coupled with visual detection. Food Control.

[31] Wang S., Liu Y., Jiao S., Zhao Y., Guo Y., Wang M., Zhu G. (2017). Quantum-dot-based lateral flow immunoassay for detection of neonicotinoid residues in tea leaves. J. Agric. Food Chem..

[32] Zhang Y., Zhang Q., Li S., Zhao Y., Chen D., Wu Y. (2020). Simultaneous determination of neonicotinoids and fipronils in tea using a modified QuEChERS method and liquid chromatography-high resolution mass spectrometry. Food Chem..

[33] Li H., Dong B., Dou L., Yu W., Yu X., Wen K., Ke Y., Shen J., Wang Z. (2020). Fluorescent lateral flow immunoassay for highly sensitive detection of eight anticoagulant rodenticides based on cadmium-free quantum dot-encapsulated nanospheres. Sensor. Actuat. B–Chem..

[34] Tian L., Guo H., Li J., Yan L., Zhu E., Liu X., Li K. (2021). Fabrication of a near-infrared excitation surface molecular imprinting ratiometric fluorescent probe for sensitive and rapid detecting perfluorooctane sulfonate in complex matrix. J. Hazard. Mater..

