# Supplementary material for: A Rapid Fluorescence Sensor for the Direct Quantification of Rongalite in Foodstuffs
Source: Foods. 2022 Sep 1;11(17):2650. doi: 10.3390/foods11172650 (PMC9455777; doi:10.3390/foods11172650)
Supplement: Supplementary file 1 [file foods-11-02650-s001.zip › foods-1871884-supplementary.pdf]

Supplementary for Foods

A rapid fluorescence sensor for the direct quantification of rongalite in foodstuffs

Hongfang Li [1, 2, *], Jie Chen[1], Baowei Huang[1], Lingwei Kong[1], Feifei Sun[2], Lin Li[2], Chuanyi Peng[1], Huimei Cai[1], Ruyan Hou[1,*]

1 State Key Laboratory of Tea Plant Biology and Utilization, College of Tea and Food Science & Tech-nology, Anhui Agricultural University, Hefei 230036, China

2 Animal-derived food safety innovation team, College of Animal Science and Technology, Anhui Agri-cultural University, Hefei 230036, China

* Correspondence: lihongfang@ahau.edu.cn; hry@ahau.edu.cn

* Author to whom correspondence should be addressed

Tel: +86 -551-65786765;

E-mail: lihongfang@ahau.edu.cn; hry@ahau.edu.cn

Figure and Table Captions

Figure S1. The HPLC-MS of dopamine hydrochloride, resorcinol and mixture solution.

Figure S2. The HPLC-MS of the mixture of dopamine hydrochloride and rongalite, the mixture of resorcinol and rongalite.

Figure S3. The fluorescence intensity at different pH values.

Figure S4. The fluorescence intensity at different carbonate solution concentrations.

Figure S5. The matrix effect of vermicelli, wheat and rice powder for the fluorescence sensor.

Figure S6: The matrix calibration curve of vermicelli, wheat and rice powder.

Table S1. An overview of the reported method for the rapid detection of rongalite.

Table S2: The limit of detection of three samples using fluorescence sensor.

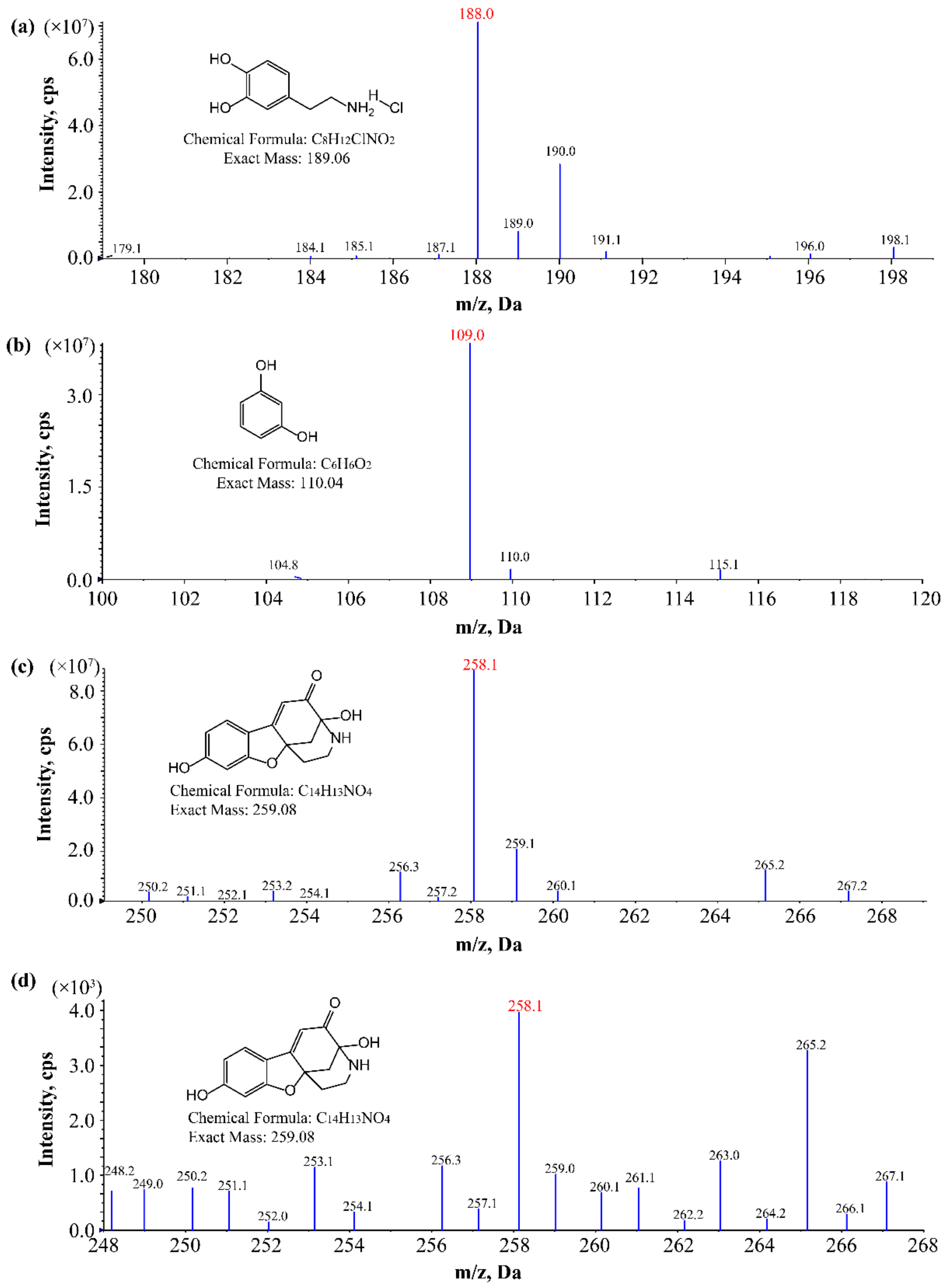

Figure S1. The HPLC-MS of (**a**) dopamine hydrochloride, (**b**) resorcinol, (**c**) reaction product of dopamine hydrochloride and resorcinol, (**d**) the mixture of rongalite, dopamine hydrochloride and resorcinol. The concentration of dopamine hydrochloride, resorcinol and rongalite was 50 μM, 50 μM and 30

μg/mL, respectively. The reaction product of dopamine hydrochloride and resorcinol and the reaction product of dopamine hydrochloride, resorcinol and rongalite were not purified before being measured using HPLC-MS and were mixture solutions.

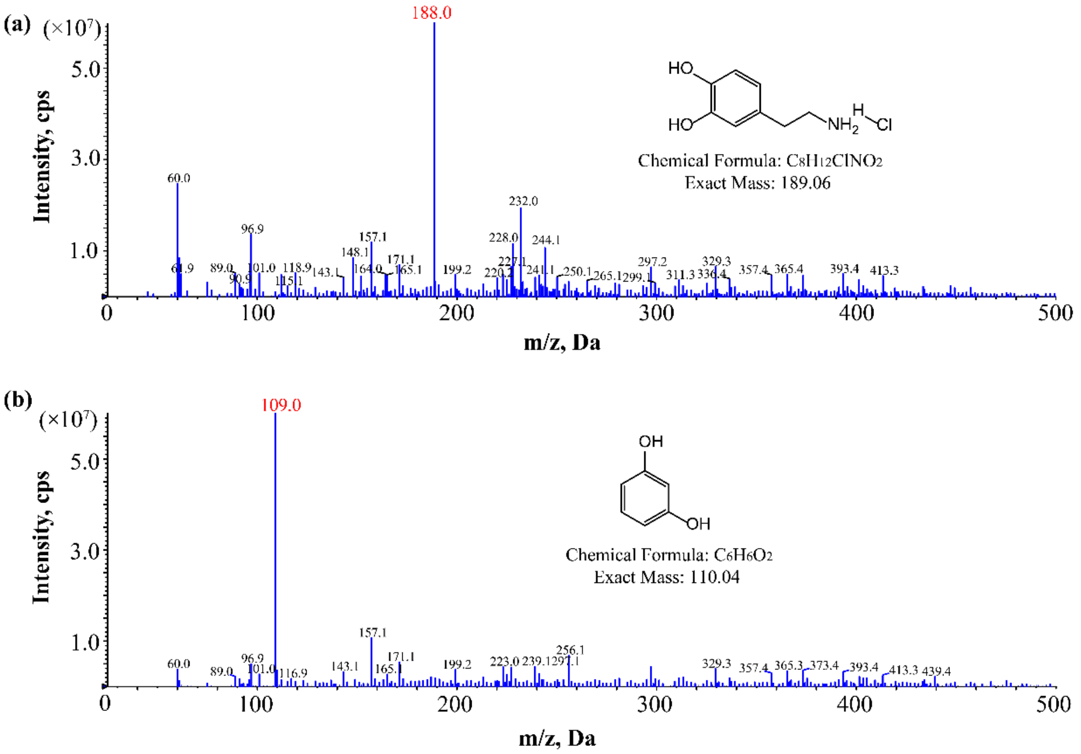

Figure S2. The HPLC-MS of (**a**) the mixture of dopamine hydrochloride and rongalite, (**b**) the mixture of resorcinol and rongalite. The concentration of dopamine hydrochloride, resorcinol and rongalite was 50 μM, 50 μM and 30 μg/mL, respectively. The mixture of dopamine hydrochloride and rongalite, and the mixture of resorcinol and rongalite were not purified before being measured using HPLC-MS and were mixture solutions.

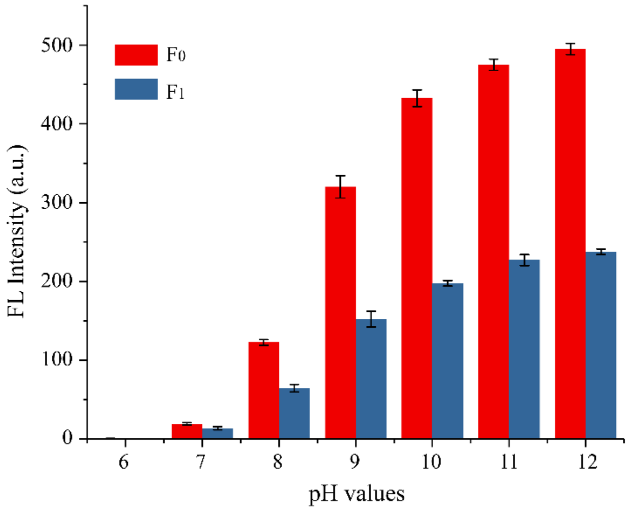

Figure S3. The fluorescence intensity at different pH values. F_0 and F_1 mean the fluorescence intensity in the absence (0 μg/mL) and in the present (0.25 μg/mL) of rongalite, respectively.

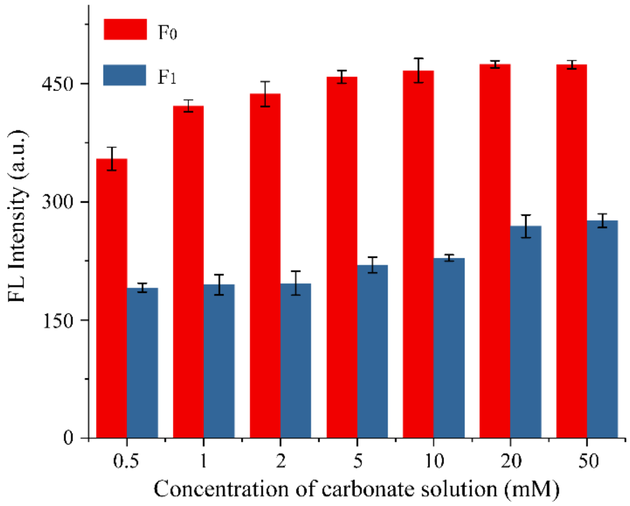

Figure S4. The fluorescence intensity at different carbonate solution concentrations. F_0 and F_1 mean the fluorescence intensity in the absence (0 μg/mL) and in the present (0.25 μg/mL) of rongalite, respectively.

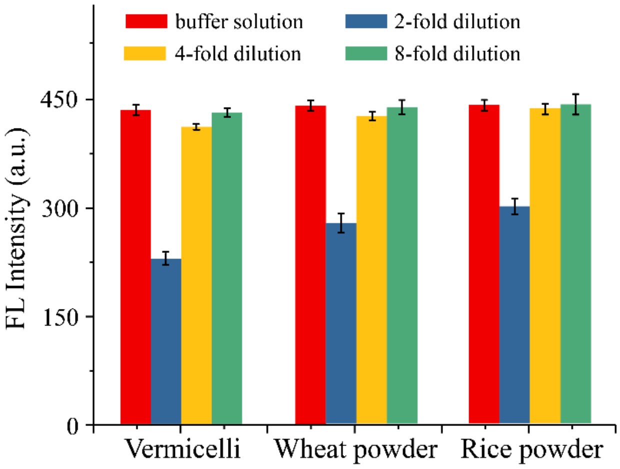

Figure S5. The matrix effect of vermicelli, wheat and rice powder for the fluorescence sensor. 2, 4 and 8-fold means the matrix solution was diluted for 2, 4 and 8 fold, respectively. All the fluorescence intensity was measured in the absence of rongalite.

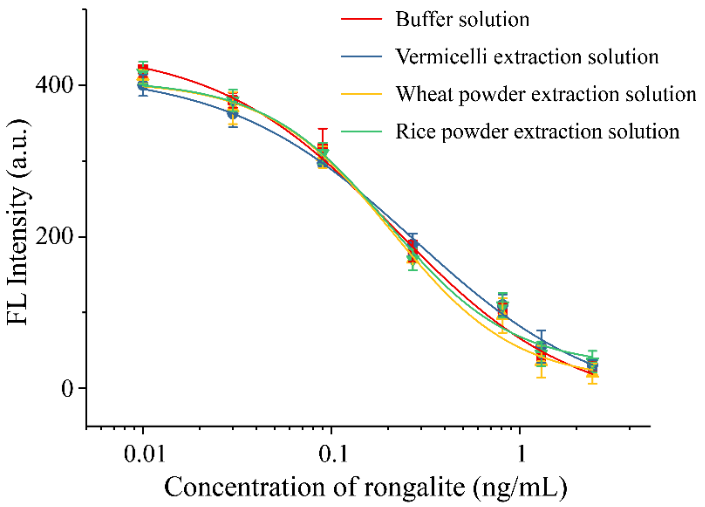

Figure S6. The matrix calibration curve of vermicelli, wheat and rice powder, respectively. The extraction solution of samples was diluted 4-fold with buffer solution and then spiked with rongalite at different concentrations.

Table S1. An overview of the reported methods for the rapid detection of rongalite.

Strategy	Material	Samples	Pretreatment of samples	Linear range (μg/mL or μg/g)	LOD (μg/mL)	Ref.
ECL[a]	$Ru(bpy)_3^{2+}$	Tofu	Samples were extracted with distilled water. The dilution factor was 40.	4.7-118	0.07	Abdussalam, et al., 2020
ELISA[b]	Aptamer	Yuba, Vermicelli, Wheatmeal, Sticky rice flour	Samples were extracted with ultrapure water. The dilution factor was 2.5-5.	-[c]	0.57×10^{-3}	Jing, et al., 2018
LFIA[d]	Aptamer	-	Samples were extracted with water. The dilution factor was 10.	-	1.0	Li, et al., 2018
Fluorescent assay	Dopamine, resorcinol	Vermicelli, wheat, rice powder	Samples were extracted with carbonate buffer solution. The dilution factor was 20.	1-18	0.28-0.38	This study

[a] means of electrochemiluminescence.

[b] means enzyme-linked immunosorbent assay.

[c] means details not shown in the article.

[d] means lateral flow immunoassay.

Table S2. Limit of detection of three samples using fluorescence sensor.

Samples	$F_{average}$ [a]	SD[b]	LOD (μg/mL)
Vermicelli	400.11	0.66	0.38
Wheat powder	405.40	1.77	0.28
Rice powder	406.35	0.31	0.32

[a] means average fluorescence intensity of 20 negative samples.

[b] means standard deviation.
